# Computational prediction of strain-dependent diffusion of transcription factors through the cell nucleus

**DOI:** 10.1007/s10237-015-0737-2

**Published:** 2015-10-17

**Authors:** Michele M. Nava, Roberto Fedele, Manuela T. Raimondi

**Affiliations:** Department of Chemistry, Materials and Chemical Engineering “Giulio Natta”, Politecnico di Milano, Piazza L. da Vinci, 32, 20133 Milan, Italy; Department of Civil and Environmental Engineering (DICA), Politecnico di Milano, Piazza L. da Vinci, 32, 20133 Milan, Italy

**Keywords:** Mechanobiology, Mesenchymal, Transcription factors, Multiphysics, Computational models

## Abstract

Nuclear spreading plays a crucial role in stem cell fate determination. In previous works, we reported evidence of multipotency maintenance for mesenchymal stromal cells cultured on three-dimensional engineered niche substrates, fabricated via two-photon laser polymerization. We correlated maintenance of multipotency to a more roundish morphology of these cells with respect to those cultured on conventional flat substrates. To interpret these findings, here we present a multiphysics model coupling nuclear strains induced by cell adhesion to passive diffusion across the cell nucleus. Fully three-dimensional reconstructions of cultured cells were developed on the basis of confocal images: in particular, the level of nuclear spreading resulted significantly dependent on the cell localization within the niche architecture. We assumed that the cell diffusivity varies as a function of the local volumetric strain. The model predictions indicate that the higher the level of spreading of the cell, the higher the flux across the nucleus of small solutes such as transcription factors. Our results point toward nuclear spreading as a primary mechanism by which the stem cell translates its shape into a fate decision, i.e., by amplifying the diffusive flow of transcriptional activators into the nucleus.

## Introduction

Cell morphology is intrinsically related to cellular functions, including proliferation, differentiation and cytoskeletal organization (Wang and Thampatty 2006, 2008; Discher et al. 2009; Hadjipanayi et al. 2009a, b; Friedman et al. 2009; Webster et al. 2009; Nava et al. 2012). Changes in the cytoskeletal architecture may induce modifications of nuclear shape, implying also variations of nuclear membrane permeability to factors involved in mechanotransduction (Dahl et al. 2008; Dupont et al. 2011; Gupta et al. 2012; Iyer et al. 2012; Schachter et al. 2012).

Several in vitro studies investigated the relationship between nuclear shape and gene expression. Among the others, Thomas et al. (2002) cultured primary osteogenic cells on microfabricated substrates with a specific surface chemistry (e.g., adhesive islands) aiming to confine cell attachment and spreading. They observed a strong correlation between nuclear spreading and gene expression for bone-specific differentiation markers (e.g., collagen I). In another contribution (Dupont et al. 2011), mesenchymal stromal cells (MSCs) were cultured on adhesive microislands. A greater amount of fluorescently labeled transcription factors was found in the nuclei of spread cells in comparison with that observed in roundish cells. On the basis of experiments involving the detachment of cells from a substrate, interpreted at the light of computer simulations, other authors (Jean et al. 2004, 2005) proved that stress states induced in the nucleus by the cytoskeletal fibers are quantitatively sufficient to alter the functions of DNA and chromatin. All these findings indicate that cytoskeletal spreading and relevant nuclear distortions may alter the import flux of transcription factors toward the nucleus and control the expression of genes and the protein synthesis.

**Fig. 1:**
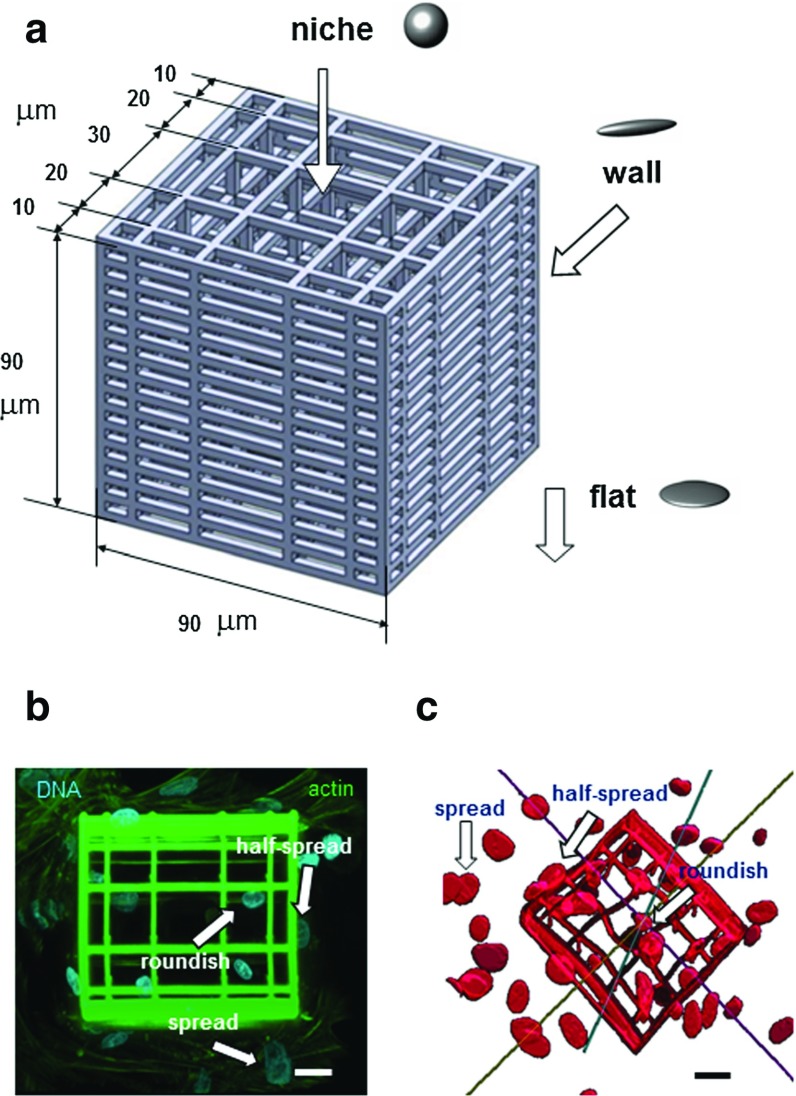
**a** The engineered niche used to induce different levels of nuclear spreading of cells cultured on the same sample. Locations of the sample where cultured mesenchymal stromal cells (MSCs) exhibited common configurations: (1) flat glass surrounding the microstructure, where spread configuration was met; (2) 3D-patterned vertical niche walls, hosting half-spread cells; (3) niche internal lattice, where cell resulted approximately roundish. **b** Confocal projection of the niche-cultured sample stained for immunofluorescence markers: DNA is stained in *blue* (DAPI); actin filaments are stained in *green*. **c** Fully 3D reconstruction of the niche cellularised sample. *Scale bars* equal $20~\upmu \hbox {m}$

In previous works (Raimondi et al. 2013, 2014), we described the fabrication process of an advanced substrate composed of three-dimensional (3D) microstructures acting as synthetic “niches” for cell culture (Fig. 1a). The niches were fabricated using the hybrid organic–inorganic material SZ2080 (Ovsianikov et al. 2008) via a direct laser writing technique, referred to as two-photon laser polymerization (2PP). We could thus observe that MSCs cultured in those 2PP niches were maintaining better multipotency, if compared to those cultured on standard flat two-dimensional (2D) glass substrates, where spontaneous differentiation occurred. We addressed this experimental response to the different configurations (i.e., different levels of nuclear spreading) promoted by the interaction of cells with the 2PP-fabricated substrate. Indeed, three main configurations of cultured cells could be distinguished on the same sample: (i) cells anchored to the flat glass surrounding the microstructures exhibited spread nuclei; (ii) cells anchored to 3D-patterned vertical niche walls possessed half-spread nuclei; and (iii) for cells anchored to the niche internal lattice, the nuclei resulted approximately roundish (Fig. 1a).

In this work, the experimental evidences arising from culturing MSCs on 2PP-engineered niches were interpreted at the light of multiphysical simulations. The main modeling assumption was that the stress states acting on the nucleus during cell adhesion induce strains which, in turn, alter locally the transport of transcription factors diffusing from the cytoplasm and involved in stem cell differentiation. The cellularized samples were imaged via confocal microscopy (Fig. 1b), and the resulting Z-stack digital images were post-processed to attain a fully 3D geometric reconstruction of the 2PP niche-cultured cells (Fig. 1c). In this way, several nuclear features could be estimated, in the aim to set up the novel strain-dependent diffusion model.

*Notation*. In what follows, the acronyms MSC and 2PP will denote the cultured mesenchymal stromal cells and two-photon polymerization, respectively. According to the tensor notation adopted hereafter, double dots will denote contraction of two inner indices (in the same order) of tensors involved, namely $\mathbf {D}{:}\mathbf {E}=D_{ijkl}\,E_{kl}$, being $\mathbf {E}$ and $\mathbf {D}$ tensors of second and fourth order, respectively. The left gradient of a vector field, the left divergence of a tensor field and the left product by the normal vector will be indicated as $\nabla \mathbf {u}=\frac{\partial \,u_{j}}{\partial \,X_{i}}$, $\nabla \cdot \mathbf {P}=\frac{\partial \,P_{ji}}{\partial \,X_{j}}$ and $\mathbf {n}\cdot \mathbf {P}=n_{j}\,P_{ji}$, respectively. Variables with pedex ${}_{0}$ concern the initial, reference configuration, which for cultured cells coincides with the roundish configuration.

## Materials and methods

### Microfabrication of the niche structures in the photoresist

The fabrication of scaffolds by 2PP was detailed in our previous work (Raimondi et al. 2013). Here let us just mention that eight niche architectures were produced using the SZ2080 photoresist (Ovsianikov et al. 2008). The niches were straight parallelepipeds, all with sizes fitting in a volume of $100 \times 100 \times 100~\upmu \hbox {m}^{3}$, constituted of an external containment grid made of horizontal parallel beams, $10~\upmu \mathrm{m}$ thick, identically spaced by $5\ \upmu \mathrm{m}$ and by an internal 3D lattice with variable geometry and size. According to their heights, niches can be first distinguished in “short,” $20~\upmu \mathrm{m}$ in height, and “tall” ones, 80–$100~\upmu \mathrm{m}$ in height. For each of the above niche typologies, we developed four alternative internal lattices, three of which had fixed pore dimension (10, 20 and $30~\upmu \mathrm{m}$), and one exhibited a pore dimension graded from 10 to $30~\upmu \mathrm{m}$ in the horizontal plane (Fig. 1a). Vertical spacing in the internal lattice was the same as the horizontal one for the uniformly spaced structures, while it was equal to $15~\upmu \mathrm{m}$ in the graded structures. The 2PP niches were directly laser written on the bottom glass wells of multiwell chambered cover glasses (Labtek, ThermoScientific-Nunc, USA).

### Cell culture

To populate the 2PP-engineered niches, primary MSCs were recovered from bone marrow by exploiting their tendency to adhere tightly to plastic culture dishes. To this purpose, 2-month-old Lewis or Sprague–Dawley rats were killed, and their femurs and tibias were aseptically removed. Bone marrow was flushed from the shaft of the bones with $\alpha$-MEM medium (Invitrogen/Gibco) containing 5 % FCS, 1 % penicillin / streptomycin and then filtered through a $100~\upmu \mathrm{m}$ sterile filter to produce a single-cell suspension. Thereafter, filtered bone marrow cells were plated in $\alpha$-MEM supplemented with 20 % FCS and 1 % penicillin/streptomycin and allowed to adhere for 24 h. Non-adherent cells were removed. The medium was changed regularly every 3 days until confluence. Adherent cells were detached by trypsin–EDTA (0.5–$0.2\hbox { g/l}$; Invitrogen), counted and cryopreserved in $\alpha$-MEM supplemented with 30 % FCS and 5 % dimethyl sulfoxide (DMSO) until use. After resuscitation, cells were plated and cultured until semi-confluence in standard flasks in complete medium. The 2PP-written chambered cover glasses were washed thoroughly, kept for 12 h in deionized water, disinfected for 12 h in 70 % ethanol, washed repeatedly in sterile deionized water, dried and UV-sterilized. For cell seeding, the cells were suspended in complete medium, counted and seeded directly in the wells of the chambered cover glasses, at a density of 20,000 cells/cm$^{2}$. The cells were incubated for 6 days with medium freshly replaced every day. All the reagents used were from Sigma-Aldrich (Germany) unless specified differently.

### Confocal imaging of cellularized samples and geometry reconstruction

To perform observations by confocal microscopy, the cells were fixed in the wells in 2 % paraformaldehyde, permeabilized with Triton 0.2 %, blocked with 3 % bovine serum albumin (BSA) plus 10 % FCS, and fluorescently marked. The cell nuclei were marked in blue using DAPI in solution at $10~\upmu \hbox {g/ml}$. Image acquisition was performed on 12 cell-populated niches. Specifically, we performed two acquisitions for the “tall” height-type (80–$100~\upmu \mathrm{m}$ height) and one acquisition for the “short” height-type niches ($20~\upmu \mathrm{m}$ height), separately for the four different internal lattices (namely with a fixed pore dimension of 10, 20 and $30~\upmu \mathrm{m}$ and one with pore dimension graded from 10 to $30~\upmu \mathrm{m}$). Sequential images, acquired in fluorescence on the DAPI channel at $1~\upmu \mathrm{m}$ intervals along the *Z* (vertical) axis, were imported in ImageJ 1.43 software (National Institute of Mental Health, Bethesda, MD, USA). Each Z-stack sequence was converted into grayscale images with 8-bit encoding. Z-stacks were preprocessed by using a median filter (connectivity of pixels equals to 4) to reduce the noise, while the remaining artifacts were removed manually. The 3D reconstruction of each Z-stack was performed by an ImageJ 1.43 plug-in (MicroSCBioJ) which is a collection of three plug-ins suitable to create and visualize 3D fluorescence volume rendering. In particular, Mesh Maker MicroSCBioJ plug-in was allowed to define the voxel dimensions and the threshold for segmentation. Resolution of each image at varying *Z* was set to $1024 \times 1024\hbox { pixels}$: the corresponding voxel dimensions were set to $0.207~\upmu \mathrm{m}$ for the *X*- and *Y*-axes, and to $1~\upmu \mathrm{m}$ along the *Z*-axis. An optimal threshold value for each Z-stack was assessed by trials. Thereafter, the regions of interest (ROIs) corresponding to the cell nuclei and to the niche structure were built by voxel growing of subsequent images. The analyzed cell population, constituted of 122 reconstructed nuclei, was subdivided into three groups on the basis of dominant configurations which also correspond to distinct culturing locations on the sample (Fig. 1b): (i) spread nuclei, anchored to the flat glass surrounding the engineered niche; (ii) half-spread nuclei, attached to the niche walls; and (iii) roundish nuclei, adherent to the niche internal lattice.

For each group, several measurements of nuclear features were performed. Different from other approaches, in which the vectorial quantities are processed on the basis of their projections onto the *X*–*Y* plane, through horizontal slices at fixed *Z* which of course do not preserve spatial orientation, the algorithm herein used allowed us to estimate accurately for the involved quantities of all the vector components and relevant angles. In fact, these parameters were computed with reference to a local Cartesian frame, with the origin at the centroid of the best-fitting ellipsoid associated with each nucleus. Through this methodology, several nuclear features could be accurately assessed and stored, namely the three semi-axes, *a*, *b* and *c* ($a>b>c$), the nuclear surface, *S*, and the nuclear volume, *V*.

### Statistical analysis

Measurements relevant to each niche architecture were gathered into three groups, corresponding to the cell localization. For each group, mean values and standard deviations were computed, and the groups were compared using one-way analysis of variance (ANOVA) for independent samples. Pairwise comparisons among the individual samples were developed through the Tukey’s HSD test on the ANOVA groups, or by the Student’s *t* test. Discrepancies among groups were considered to be significant if the *p* value was not $>0.01$.

### Mathematical model and boundary conditions

The problem in point was modeled in a multiphysical framework. A spherical cell was assumed as the problem domain ${\varOmega }$, constituted of cytoplasm material and the concentric nucleus at this interior. This assumption is well corroborated by experimental observations. In fact, a spherical nuclear morphology was documented for suspended cells (Jean et al. 2004), as well as for cells anchored on substrates with a relatively low stiffness (Jacot et al. 2010) or cultured on substrates that confine the attachment and spreading of cells (Thomas et al. 2002). In the presence of large strains, a total Lagrangian formulation was adopted, i.e., all quantities were referred to the spatial coordinates in the initial configuration. Herein the two simplified phases (cytoplasm and nucleus) were assumed to behave as homogeneous and isotropic hyper-elastic materials. Their constitutive relationships, derived from a potential energy, were expressed as $\mathbf {S}=\mathbf {D}{:}\mathbf {E}$, being $\mathbf {S}$ the second Piola–Kirchhoff stress, $\mathbf {E}=\frac{1}{2}(\mathbf {F}^{T}\mathbf {F}-\mathbf {I})$ the Green–Lagrange strain, $\mathbf {F} = \nabla \mathbf {u}+\mathbf {I}$ the deformation gradient, $\mathbf {u}$ the displacement field and $\mathbf {D}$ the isotropic stiffness tensor function of the two Lamé elastic moduli. As for the mechanical problem, the conservation equation of linear momentum was formulated as follows:

1 $$\begin{aligned} \rho _{0} \frac{\partial ^{2} \mathbf {u}}{\partial t^{2}} = \nabla \,\cdot \, \left( \mathbf {S} \mathbf {F}^{T}\right) + \rho _{0} \mathbf {B} \end{aligned}$$

being $\nabla \cdot$ the (left) divergence operator, *t* the physical time, $\rho _{0}$ the initial density per unit volume and $\mathbf {B}$ the unit mass force vector (Belytschko et al. 2000). The product $\mathbf {S}\,\mathbf {F}^{T}$ represents the so-called nominal or first Piola–Kirchhoff stress tensor $\mathbf {P}$, which is not symmetric. On the contrary, from the conservation of angular momentum, second Piola–Kirchhoff stress can be proved to be symmetric, namely $\mathbf {S}=\mathbf {S}^{T}$. Perfect adhesion was considered at the cytoplasm–nucleus interface, across which jumps of traction vector $\mathbf {t}_{\mathrm {n}}=\mathbf {n}\cdot \mathbf {P}$ are not allowed, being $\mathbf {n}$ the outward normal referred to the initial configuration. Since Eq. 1 is second order in time, initial conditions on both displacement and velocity fields over the whole domain ${\varOmega }$ were specified. To simulate the volumetric change of the nucleus that occurs during cell spreading and anchoring on a flat substrate, we prescribed monotonically increasing displacements (or, equivalently, constant velocities) over a part of the cell boundary (Dirichlet boundary conditions) and traction-free conditions (Neumann ones) over the complementary outer frontier. By symbols, one has $\mathbf {t}_{\mathrm {n}}=\mathbf {n}\cdot \mathbf {P}=\mathbf {0}$ over $\partial {\varOmega }_{t}$ and $\mathbf {u}=\overline{\mathbf {u}}(t)$ over $\partial {\varOmega }_{u}$, where $\partial {\varOmega }_{u}\bigcup \partial {\varOmega }_{t}=\partial {\varOmega }$, $\partial {\varOmega }_{u}\bigcap \partial {\varOmega }_{t}=\{\emptyset \}$.

Passive diffusion of transcription factors toward the nucleus was modeled by the following equation:

2 $$\begin{aligned} \frac{\partial {c}}{\partial {t}} = \nabla \,\cdot \, (\,D\, \nabla c\,) \end{aligned}$$

where symbols *D* and *c* denote the molar diffusion coefficient and the molar concentration of transcription factors, respectively, being ${\varvec{\phi }}=-D\,\nabla c$ the molar diffusive flux according to the first Fick’s law. Equation 2, often referred to as second Fick’s law, assumes that the local rate of change of concentration *c* is approximately proportional to the second space derivatives of the concentration itself (i.e., to its “curvature”), although a space varying diffusivity *D* may modulate this relationship. The above parabolic equation was endowed by an initial condition (at $t=0$) on molar concentration *c* over ${\varOmega }$, as well as by conditions on vanishing diffusive flux (${\varvec{\phi }}=\mathbf {0}$) over the outer boundary $\partial {\varOmega }$ at varying time *t*, describing complete insulation. Moreover, continuity for the same flux was prescribed over the inner cytoplasm–nucleus interface. It is worth noting that the diffusion coefficient *D* in Eq. 2 may exhibit dependence also on the molecular weight of the solute.

To couple the mechanical problem and the diffusion problem in Eqs. 1 and 2, respectively, we introduced the following closed-form dependence of the molar diffusion coefficient on deformation field (Klepach and Zohdi 2014):

3 $$\begin{aligned} D(J)= D_{0} \,\,\frac{e^{\,J}-1}{e-1} \end{aligned}$$

Symbol $J=\mathrm {det}(\mathbf {F})$ denotes as usual the Jacobian determinant, from which one can compute the volumetric strain $\varepsilon _{V}$= $J-1$ and the current density through mass conservation $\rho \,J=\rho _{0}$. Coefficient $D_{0}=D(1)$ has to be calibrated on the basis of experimental data.

Although Eq. 3 has been proposed with reference to a generic heterogeneous medium and not specifically for biological tissue, the behavior indicated by such simple but effective formula is rather intuitive. In fact, under compressive stress states, diffusivity is expected to vanish, the medium becoming fully densified. On the contrary, when the nuclear volume ratio increases ($J > 1$) consistently with experimental observations reported in Figs. 4 and 5, a condensation of matter, which acts as a diffusion barrier, occurs and the spatial distribution of diffusivity is expected to change and achieve larger (or lower) values than that of the reference (i.e., roundish) configuration. From a mathematical standpoint, this circumstance implies that the following conditions hold: $D(0) = 0$, $D (1) = D_{0}$. The adopted formulation of Eq. 2 does not include the additional terms which refer the jacobian-dependent diffusivity *D* to the initial configuration, see (Federico et al. 2015). The experimental information available so far was not sufficient to identify the “actual” functional dependence of diffusivity on *J* or on other field variables (e.g., on the concentration itself), and Eq. 3 has to be regarded as an a priori information. It is worth emphasizing that, in the mechanical problem governed by Eq. 1, the rate of prescribed boundary displacement was selected on the basis of experimental and mechanical considerations, to be critically discussed in Sect. 4.

### Physical and mechanical parameters

**Fig. 2:**
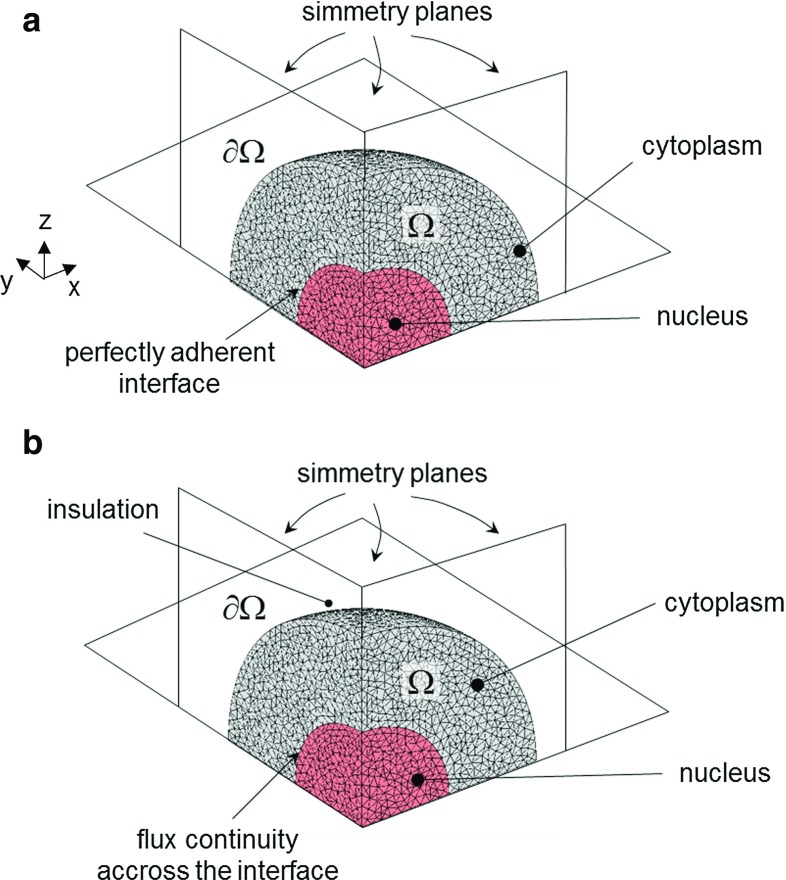
Model geometry and boundary conditions for the mechanical problem, in (**a**), and the diffusion problem, in (**b**)

Equations 1 and 2, governing in a continuum medium the mechanical and diffusion problems, respectively, coupled via Eq. 3, can be given a weak formulation through rather standard approaches, herein not reported for brevity. The commercial software Comsol $\hbox {Multiphysics}^{\circledR }$ (Comsol 2008) was used to discretize in a finite element framework and numerically solve the above coupled problems and to post-process the results. More specifically, in the programming platform offered by $\hbox {Comsol}^{\circledR }$ the structural mechanics and the mass transport modules were utilized, while the coupling constraint specified by Eq. 3 was introduced by the user. Symmetries were exploited for both the mechanical and the diffusion problems, so that the numerical analyses were limited to one octave of the whole spherical cell (Fig. 2). The problem domain ${\varOmega }$ was discretized by means of 98,000 10-node quadratic tetrahedrons. We used the default solver settings arisen from the commercial software. The time integration algorithm was implicit by variable-order variable-step-size backward differentiation formulas (the Comsol option for the integration time step was set to “Strict”). The inner sphere of radius $5~\upmu \mathrm{m}$ represents the nuclear domain, while the outer part inside the sphere of radius $10~\upmu \mathrm{m}$ represents the cytoplasm domain. The Young’s modulus *E* for the nucleus and the cytoplasm were set to 5 and 1 kPa, respectively (Vaziri and Mofrad 2007). To take into account both the fluid-like (almost isochoric) and the solid-like (compressible) behavior of the cytoplasm and of the nucleus, consistently with the novel experimental data available on nucleus volume change (see Figs. 4, 5), the Poisson’s ratio $\nu$ for the two phases was set to 0.35 (Jean et al. 2004; Vaziri et al. 2006). This assumption for the nucleus is consistent with experimental observations on isolated endothelial cell nuclei. Regarding the diffusive transport problem, the molar diffusion coefficient $D_{0}$ in Eq. 3 was set to $30.0~\upmu \mathrm{m}^2/\hbox {s}$ for the cytoplasm and to $29.2~\upmu \mathrm{m}^2/\hbox {s}$ (Kuhn et al. 2011) for the nucleus. At instant $t=0$, the transcription factors were entirely concentrated in the cytoplasm, for which an initial value of molar concentration *c* was assumed equal to $0.2~\upmu \mathrm{m}\hbox {ol/ml}$. Experimental studies, see, e.g. (Dupont et al. 2011), confirm that the concentration of mechanobiological transcription factors within the nuclei of roundish cells is negligible.

## Results and discussion

### Measurements of nuclear features

**Fig. 3:**
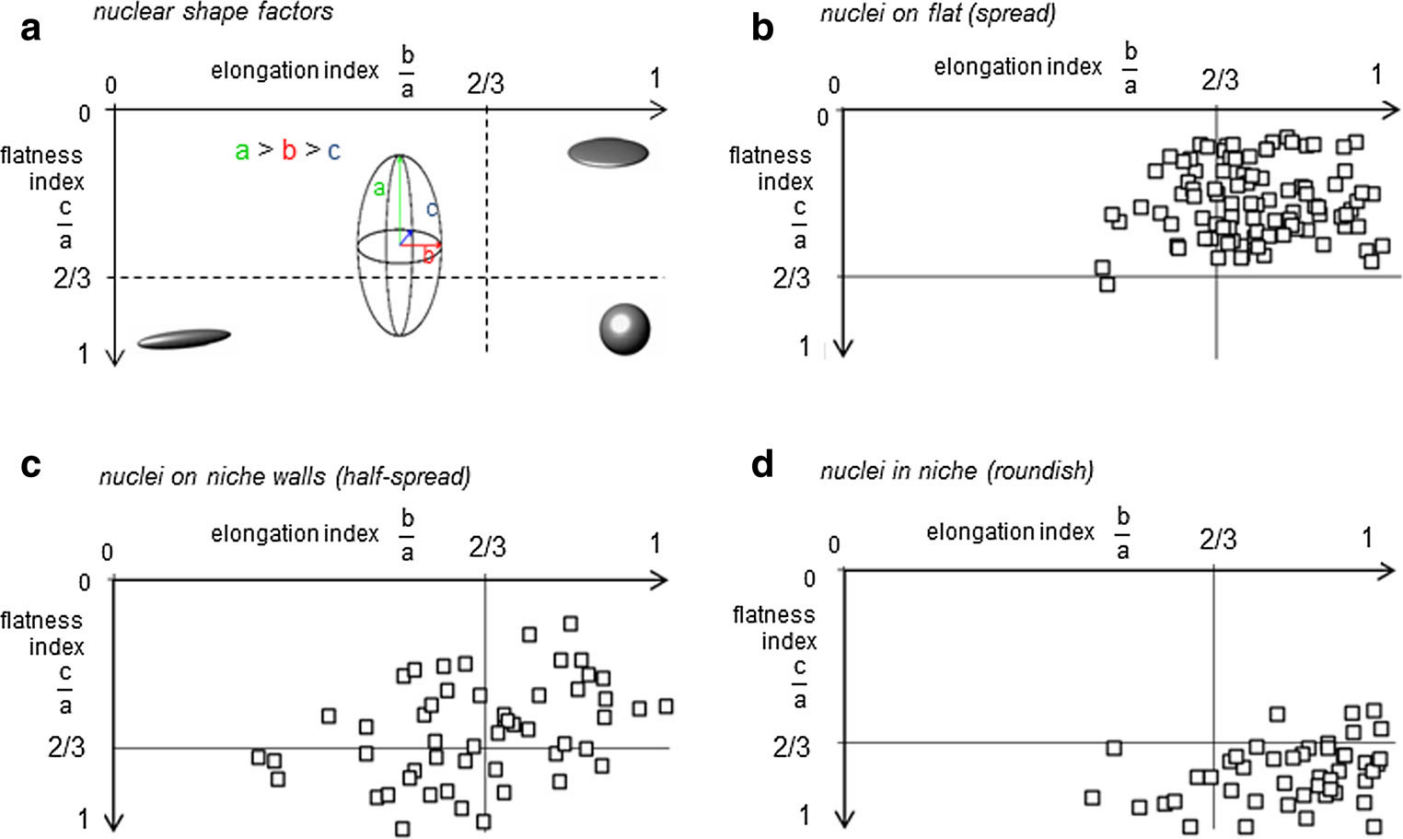
Visualization of the nuclear geometry in terms of elongation index *b* / *a* versus the flatness index *c* / *a*, being *a*, *b* and *c* the semi-axes of the best-fitting ellipsoid sketched in (**a**). Measurements of spread cell nuclei anchored to the flat glass surrounding the engineered niche, in (**b**); half-spread cell nuclei, attached to the niche walls, in (**c**); roundish cell nuclei adherent to the niche internal lattice, in (**d**)

**Fig. 4:**
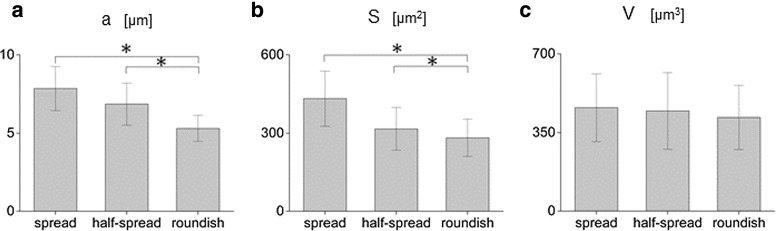
Measurements of nuclear features for spread, half-spread and roundish nuclei, in terms of best-fitting ellipsoid parameters: major semi-axis *a*, in (**a**), surface *S*, in (**b**), volume *V*, in (**c**). Histograms of mean values endowed by 99 %-confidence bars were derived from population of 122 MSCs. Symbol *asterisks* indicates statically meaningful differences, with *p* values not $>0.01$ for all pairwise comparisons

**Fig. 5:**
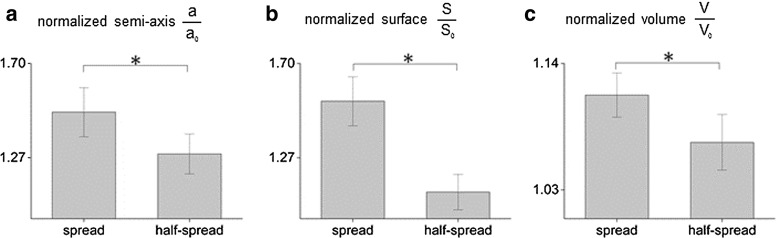
Measurements of nuclear features for spread and half-spread nuclei as in Fig. 4, normalized with respect to their counterparts in the roundish configuration, denoted by pedex ${}_{0}$

To perform a quantitative assessment of nuclear geometry, a population of 122 cells was monitored by means of confocal microscopy. As already mentioned, cell nuclei assumed dominant configurations depending on their location in the 2PP-niche sample. As meaningful shape factors for these nuclei, we introduced the elongation and the flatness indices, defined as *b* / *a* and *c* / *a*, respectively, where *a*, *b*, *c* are the semi-axes of the best-fitting ellipsoid, obeying to the condition $a>b>c$ (Fig. 3a). As shown in Fig. 3b, spread nuclei outside the niche possessed an elongation index *b* / *a* close to unity and a flatness index *c* / *a* less than 2 / 3 indicating a disk-like shape. Among them, very few nuclei exhibited a rod-like shape (Fig. 3b). Highly scattered elongation and flatness ratios were observed for half-spread nuclei (see Fig. 3c) adhered to the niche-containing walls, while roundish nuclei inside the niche showed a spherical morphology with almost unitary values of both indices (Fig. 3d). The nuclear features derived by confocal images on the 2PP niche are synoptically outlined in Fig. 4. The major semi-axis *a*, measured in spread and half-spread cells, resulted $7.89\pm 1.41$ and $7.85 \pm 1.30~\upmu \mathrm{m}$, respectively, being the mean values endowed by 99 % quantiles as confidence intervals. Both values are significantly larger than the roundish cell counterpart, which amounts to $5.30\ \pm \ 0.73~\upmu \mathrm{m}$ (Fig. 4a). Consistently, the nuclear surface *S*, calculated for spread ($417.74 \pm 125.01~\upmu \mathrm{m}^2$) and half-spread cells ($316.28 \pm 82.75~\upmu \mathrm{m}^2$), turns out to be significantly larger than those observed for the roundish configuration ($282.42 \pm 71.77~\upmu \mathrm{m}^2$), see also Fig. 4b. On the contrary, discrepancies among spread, half-spread and roundish nuclear volumes *V* were small and close to the current measurement accuracy (Fig. 4c). These findings confirm the effectiveness of the proposed niche culture system in promoting different nuclear shapes and therefore distinct levels of cell spreading, guided by the adhesive interactions between the cell and the substrate. Moreover, the above-mentioned geometrical features for spread and half-spread nuclei were normalized by their averaged counterparts detected for the roundish nuclei, denoted in what follows by pedex ${}_{0}$, leading to the non-dimensional ratios $a/a_{0}$, $S/S_{0}$ and $V/V_{0}$ for the major semi-axis, the nuclear surface and the volume, respectively, visualized in Fig. 5. Both major semi-axis and surface of fitting ellipsoids for spread nuclei were 1.6 times larger than their counterparts in roundish nuclei. Volumes of cell nuclei outside the grid and those anchored at the lateral walls of the culture niches resulted 1.3 and 1.12 times respectively larger than those lying in the niche inner lattice (Fig. 5a, b). Moreover, an increase of about 10 % was observed for the cell volumes when passing from roundish to spread configurations (Fig. 5c). All these findings suggest that different culture locations inside the niche favor different levels of nuclear spreading, ultimately depending on the mutual interactions between the cell and the substrate. With respect to the roundish configuration, nuclear volumes for spread and half-spread cells resulted 10 and 8 % greater, respectively (Fig. 5c). This circumstance might be explained by the coexistence of solid-type (DNA/chromatin) and liquid-type constituents inside the nuclei (Jean et al. 2004). Such volume changes observed in cultured cells suggested to take into account nucleus compressibility in the mechanical model, expected to modulate the mass transport diffusion across the nuclear membrane.

### Model predictions

**Fig. 6:**
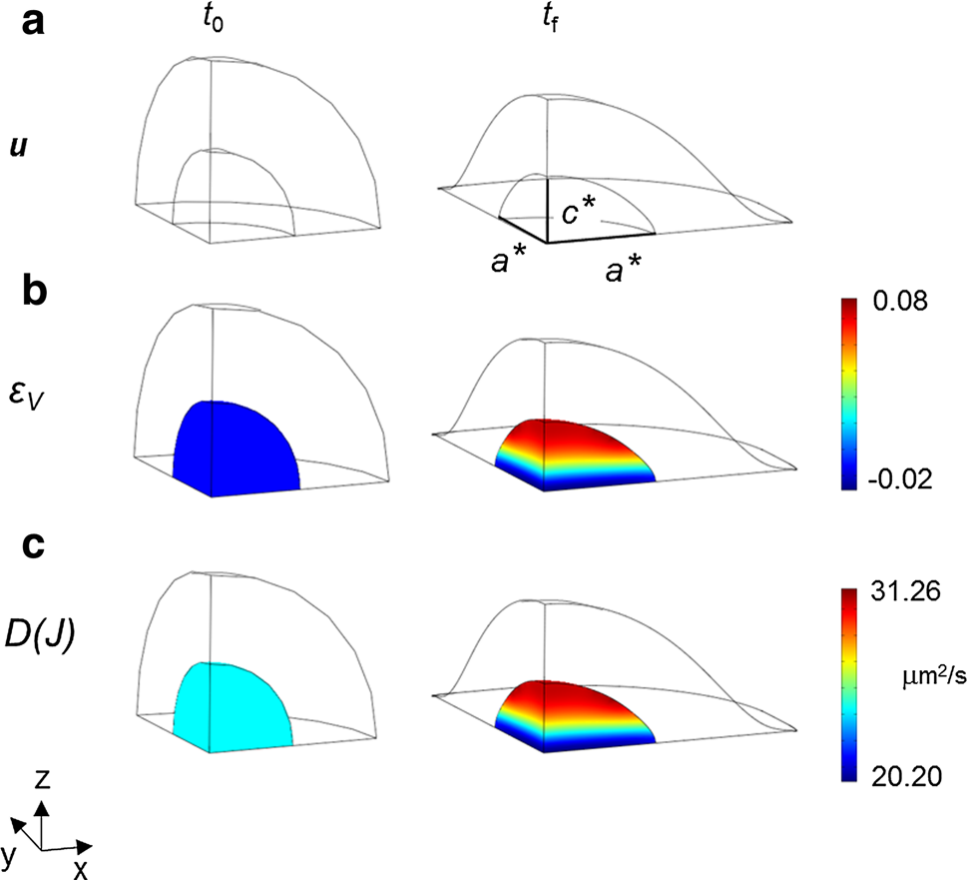
Predictions provided by the multiphysical model at the initial instant $t_{0}$ and at the final one $t_{f}=1$ s, when passing from the roundish to the spread configuration. *Contour plots* inside the nucleus of: **a** displacement field $\mathbf {u}$, computed when a uniform displacement $a^{*}$ is prescribed along the equatorial parallel at $Z=0$; **b** volumetric strain $\varepsilon _{V}$; **c** strain-dependent diffusion coefficient *D*(*J*). Prescribed nucleus length $a^{*}$ was computed by averaging major and intermediate semi-axes *a* and *b* of spread cell ellipsoids, while the length $c^{*}$ along the *Z*-axis provided by the finite element analysis significantly exceeds its experimental counterpart

To simulate the effects of cell spreading on the passive diffusion of transcription factors, the multiphysical simulations were driven by displacements monotonically increasing in time, prescribed at the equatorial parallel of the cell outer boundary (at $Z=0$, exclusively in the first octant). A first approximation consisted of prescribing a uniform (at varying the azimuthal angle in the horizontal plane) radial displacement, till the inner nucleus attained along both the *X*- and *Y*-axes the overall length $a^{*}$, see Fig. 6. Value $a^{*}=4~\upmu \mathrm{m}$ was computed by averaging the values of major and intermediate semi-axes *a* and *b* of the ellipsoids fitting the spread cells ($a > b > c$), being $10~\upmu \mathrm{m}$ the cell overall radius. The assigned deformation was prescribed at a constant rate over a duration of $t_{f}=1$ s, to be discussed later. Under such assumptions, the multiphysical response of the entire spherical cell (or exclusively of its part in the first octant) could be equally computed by means of an axial-symmetric model. As expected, the CPU time for the axial-symmetric simulation (based on 22,200 quadratic triangles) resulted 10 times lower with respect to fully 3D analyses. Moreover, the same simulation predicted that the volumetric strain $\varepsilon _{V}$ attained its maximum at the top of the nucleus, equal to about 8 % (Fig. 6b), in agreement with the experimental observations (Fig. 4c). Nevertheless, the adoption of a uniform radial displacement along the outer boundary equatorial parallel led to poor predictions of the nucleus deformation. In fact, the nucleus length $c^{*}$ along the *Z*-direction provided by the finite element analysis significantly exceeded its experimental counterpart.

For the above reasons, an alternative strategy was exploited. The radial displacement was assumed to smoothly vary along the equatorial parallel of the cell outer boundary when passing from the *X*- to the *Y*-axis, at varying the azimuthal angle in the horizontal plane (for $Z=0$). This problem cannot be modeled under axial symmetry conditions, but requires a fully 3D discretization. In this way, the values of major and intermediate semi-axes *a* and *b* observed experimentally (via best-fitting ellipsoids on confocal images) were attained with accuracy by the mathematical model at the nucleus–cytoplasm interface. Moreover, the final length §$c^{}$ of the spread nucleus along the vertical direction resulted closer to the experimental values, and the prediction error on it was drastically decreased with respect to the previous model and boundary conditions. In the authors’ opinion, this last approximation, which simulates nuclear spreading by prescribing a radial displacement to the cell varying with the azimuthal angle, has to be regarded as an optimal compromise between conflicting requirements of low complexity and prediction accuracy.

**Fig. 7:**
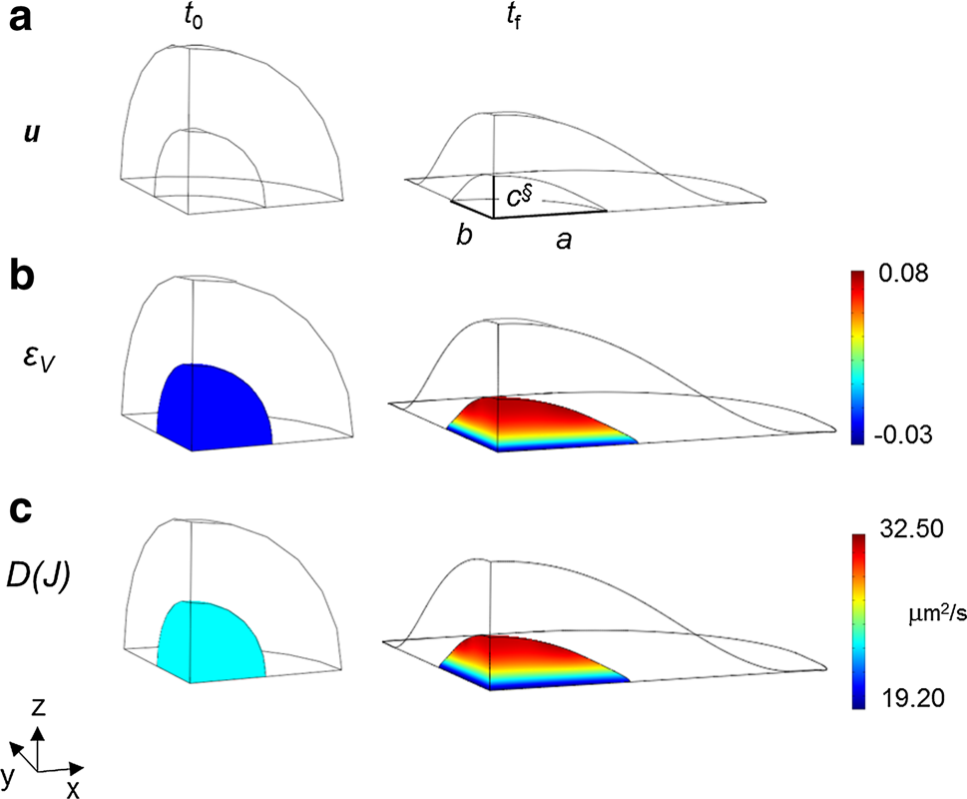
Model predictions provided by radial boundary displacement smoothly varying along the equatorial parallel at $Z=0$, with final lengths of the nucleus along the *X*- and *Y*-axis equal to major and intermediate ellipsoid semi-axes *a* and *b*, respectively, being the loading duration equal to $t_{f}=1$ s: same illustrations as in Fig. 6. Final length §$c^{}$ of the nucleus provided by the finite element analysis results significantly closer to the experimental counterpart, if compared to value $c^{*}$ of Fig. 6

**Fig. 8:**
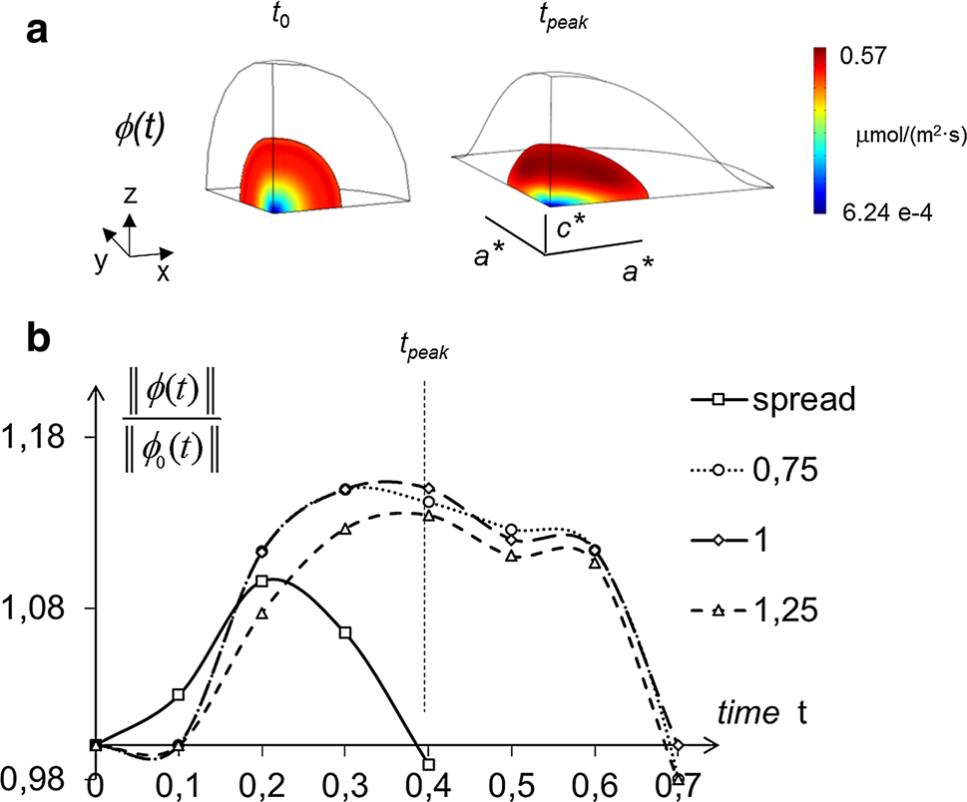
Model predictions provided by a uniform boundary displacement with final radial length of the nucleus equal to $a^{*}$, being $c^{*}$ the resulting length along the *Z*-axis: **a** *contour plot* of diffusive flux in the nucleus, at the initial instant $t_{0}$ and at instant $t_{\mathrm {peak}}$; **b** maximum modulus of diffusive flux $\varvec{\phi }$ in spread cell nuclei as a function of time, normalized by its counterpart $\varvec{\phi }_{0}$ in roundish cell nuclei, attaining its maximum at instant $t_{\mathrm {peak}}$. The *continuous line* denotes the diffusion kinetics when the mechanical deformation precedes any import of small molecules. The *dashed lines* refer to the diffusive transport starting simultaneously with the mechanical deformation, at varying rates of the prescribed displacement. *Circular*, *square* and *triangular markers* denote normalized fluxes under amplitudes of the ascending ramp equal to 0.75, 1.0 and 1.25 s, respectively

**Fig. 9:**
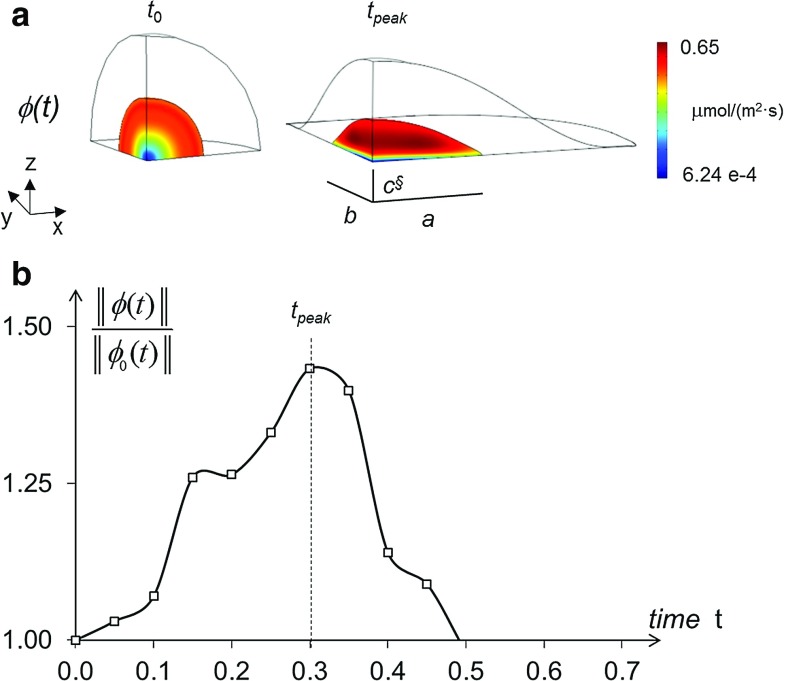
Model predictions provided by radial boundary displacement smoothly varying along the equatorial parallel at $Z=0$ (1 s ramp), with final lengths of the nucleus along the *X*- and *Y*-axis equal to major and intermediate ellipsoid semi-axes *a* and *b*, respectively, being §$c^{}$ the length along the *Z*-axis resulting from the analysis: same illustrations as in Fig. 8

The main results provided by such 3D multiphysical model are visualized in Fig. 7. When passing from the roundish to the spread configuration (Fig. 7a), the volumetric strain $\varepsilon _{V}$ attained values around 8 % along a parallel at approximately $+65^{\circ }$ (degrees) latitude, at the nucleus–cytoplasm interface: the absolute maximum was attained closely to the *X*-axis, where the nucleus length was equal to the major semi-axis *a* of fitting ellipsoid. In the same cell location, the diffusion coefficient *D*(*J*) attained its maximum, amounting to $32.50~\upmu \mathrm{m}/\hbox {s}^2$ (Fig. 7c) and resulting slightly higher then its counterpart of Fig. 6c, predicted by the equivalent axisymmetric analysis. Figure 8a shows the contour plot of diffusive flux ${\varvec{\phi }}$ in modulus, for the roundish and the spread configuration, at instant $t_{0}$ and $t_{f}$, respectively, while Fig. 8b visualizes as a function of time the same quantity at the point of maximum in the spread configuration (along the parallel mentioned above), normalized by its (time-varying) counterpart $\Vert {\varvec{\phi }}_{0}\Vert$ in a roundish configuration. In Fig. 8b, the plot of interest, consistent with a 1-s duration of deformation history considered so far, is denoted by the dashed line marked by squares. In truly 3D analyses, the normalized flux achieved a peak of about 1.40 (Fig. 9) and smoothly decreased after the spread configuration was attained. This circumstance implies that the nuclear–cytoplasm import through passive diffusion is strongly enhanced due to the nuclear spreading. To confirm the effectiveness of the present approximation with respect to that based on an equivalent uniform displacement (i.e. axisymmetric), for the latter much lower peaks of the normalized fluxes (${<}1.13$) can be observed in Fig. 8b, to be compared with plot in Fig. 9b.

## Critical remarks and future prospects

In this study, we developed a mathematical model to quantitatively assess the influence of cell spreading on the passive diffusion of mechanobiological transcription factors, directed from the cell cytoplasm into the cell nucleus. From the literature (e.g., Pajerowski et al. 2007), we already know that nuclei in stem cells are more deformable than in differentiated cells, likely due to a lower content in the nuclear structural protein lamin A (Swift et al. 2013). Also, important studies (Dupont et al. 2011; Gupta et al. 2012; Schachter et al. 2012) reported for stem cells cultured in vitro a prompt import of transcription factors into the nucleus in conjunction with cell spreading. However, the mechanisms governing this import are still poorly understood. We hypothesize that the flux of transcription factors in the cell can be altered by a strain-dependent diffusion coefficient.

It is worth emphasizing that exclusively the passive diffusion of transcription factors was considered herein, neglecting for instance the macromolecular cargo-mediated transport and focusing on instantaneous variations in the nuclear concentration of such small factors with cell configuration changes. On the contrary, intracellular cargoes are known to translocate into the nucleus at a lower speed and therefore are suitable to provide a fine regulation of gene expression (Lott and Cingolani 2011).

In Wang et al. (2009), it was stated that several seconds are required for a single cell to complete the process of passive diffusion of small molecules, including transcription factors. Among other causes affecting this time lapse, the heterogeneity of the cytoplasm (with embedded organelles) and its viscosity should be mentioned. In the present model, the cytoplasm and the nucleus were described as homogeneous media, each one with a uniform initial concentration: consistently, herein lower characteristic times are expected, in the order of a few seconds. In the roundish cell configuration, assuming a unidimensional diffusion process along the radius, the characteristic time can be estimated as $R^{2}/D$, being *R* the nuclear radius and *D* the diffusivity, respectively. This value amounts to 0.83 s for the present simulations.

For a more complete assessment, two different scenarios were compared on the basis of additional numerical analyses. (i) Firstly, we assumed that the nucleus deformation was exhausted before ($t<t_{0}$) diffusion of small molecules occurred ($t\ge t_{0}$). This circumstance is very unlike to occur, since it would imply, during the whole deformation process, a vanishing molecule flow through the nuclear membrane. Consistently with this hypothesis, however, diffusion kinetics was computed with a not homogeneous field of diffusivity *D* resulting through Eq. 3, once the final Jacobian distribution was provided by the mechanical analysis. (ii) Secondly, as hypothesized so far, we assumed that the mechanical deformation and the diffusion process started simultaneously at instant $t_{0}$, but different rates were considered for the prescribed displacement in the mechanical problem, with amplitudes of the ascending ramp equal to 0.75, 1.0 and 1.25 s. We observed that the characteristic times of passive diffusion remained approximately unchanged for these three deformation rates, as visualized in Fig. 8. On the contrary, the normalized flux computed under the hypothesis (i) at the top of the nucleus exhibited a markedly smaller peak value, and also the overall kinetics resulted more rapid. Furthermore, from the literature we know that in the cell mechanochemical conversions induced by cytoskeletal connections are extremely rapid. For instance, Na et al. (2008) reported a time of 0.3 s for the focal activation of Src kinase, even in locations distant from the site of force application (${>}50~\upmu \mathrm{m}$). In conclusion, the duration of 1 s, adopted as a methodological reference for the deformation history of the nucleus, is well rooted in experimental evidences and corroborated by mechanical considerations. The fundamental signaling event, which activates stem cell differentiation, is expected to coincide with a massive increase in the simultaneous flow of several mechanoregulatory factors, inducing a stimulus called “master switch.” Our model reproduces and quantifies very well this condition, as indicated by the peak in the spreading-dependent diffusion flow reported in Fig. 8.

To increase the predictive capabilities of the present model, a few limitations of this work should be addressed in future studies. Among others: (i) from the experimental standpoint, confocal images capture diverse cells frozen in different configurations, while the model predictions concern an individual cell deforming along time; (ii) as for the modeling assumptions, we neglected the presence of subcellular constituents, such as the cytoskeleton and the cortical later, in favor of only the two homogeneous phases, the nucleus and the cytoplasm. Spread configuration, experimentally assessed through confocal microscopy, was induced through displacements prescribed along the equatorial parallel of the outer cell boundary. To overcome difficulties related to point (i), we are planning to assess the flux of fluorescently labeled transcription factors directly on live deforming cells. As for point (ii), a straightforward comparison with literature data can corroborate the effectiveness of the present approach. In the important contribution by Jean et al. (2005), cell model included all the major subcellular structures (nucleus, cytoskeleton, cytosol, cortical layer). To describe cytoskeletal fibers associated with nucleus, they introduced reactive forces acting on the nucleus outer boundary, at uniformly distributed discrete locations. Despite its simplicity, our model was able to predict within the nucleus an average stress along the major and minor axes of the fitting ellipsoid of about 2200 and 1818 Pa, respectively, in satisfactory agreement with values of 2000 Pa reported for both the stress components in Jean et al. (2005). For the in-plane shear stress, we found a value of 370 Pa, while 500 Pa were computed by the other model.

Despite the limitations mentioned above and other simplifying assumptions, the results outlined in this paper have to be considered as rather innovative and confirm the important contribution of nuclear mechanics in mechanobiology studies. The diffusive flux predicted across the nucleus–cytoplasm interface turned out to be accelerated by the nuclear spreading, exceeding of important 40 % its counterparts in roundish nuclei. Such intensification of small molecule flux toward the nucleus can be crucial to drive stem cell fate decision. This result might also explain, by contrast, the decrease in multipotency maintenance and spontaneous commitment toward the osteochondral lineage that we observed in cells cultured on flat glass substrates (Raimondi et al. 2013, 2014). Combining synergistically our experimental measurements with finite element simulations, it can be stated that the deformation of the nucleus from a roundish to a spread configuration is a primary determinant of an important increase in diffusion fluxes of transcription factors from the cell cytoplasm to the nucleus. Future work will concern the mathematical modeling of the nuclear membrane and modifications in nuclear envelope diffusivity through the changes in the nuclear pore complex, the analytical dependence of diffusivity on local volumetric strain and possibly other field variables (such as the molar concentration), and the analysis of cultured cells through digital image correlation (Fedele et al. 2013a, b) of suitable time sequences of confocal images, in order to develop finite element model updating procedures (Fedele 2015).
